# Serum Levels of Caspase-Cleaved Cytokeratin-18 and Mortality Are Associated in Severe Septic Patients: Pilot Study

**DOI:** 10.1371/journal.pone.0109618

**Published:** 2014-10-07

**Authors:** Leonardo Lorente, María M. Martín, Agustín F. González-Rivero, José Ferreres, Jordi Solé-Violán, Lorenzo Labarta, César Díaz, Alejandro Jiménez, Juan M. Borreguero-León

**Affiliations:** 1 Intensive Care Unit, Hospital Universitario de Canarias, La Laguna, Tenerife, Spain; 2 Intensive Care Unit, Hospital Universitario Nuestra Señora Candelaria, Santa Cruz Tenerife, Spain; 3 Laboratory Department, Hospital Universitario de Canarias, La Laguna, Tenerife, Spain; 4 Intensive Care Unit, Hospital Clínico Universitario de Valencia, Valencia, Spain; 5 Intensive Care Unit, Hospital Universitario Dr. Negrín, Las Palmas de Gran Canaria, Spain; 6 Intensive Care Unit, Hospital San Jorge, Huesca, Spain; 7 Intensive Care Unit, Hospital Insular, Las Palmas de Gran Canaria, Spain; 8 Research Unit, Hospital Universitario de Canarias, La Laguna, Tenerife, Spain; University of Leicester, United Kingdom

## Abstract

**Objective:**

Apoptosis is increased in sepsis. Cytokeratin 18 (CK-18), a protein of the intermediate filament group present in most epithelial and parenchymal cells, is cleaved by the action of caspases and released into the blood as caspase-cleaved CK (CCCK)-18 during apoptosis. Circulating levels of CCCK-18 have scarcely been explored in septic patients. In one study with 101 severe septic patients, the authors reported higher serum CCCK-18 levels in non-survivors than in survivors; however, the sample size was too small to demonstrate an association between serum CCCK-18 levels and early mortality and whether they could be used as a biomarker to predict outcomes in septic patients. Thus, these were the objectives of this study with a large series of patients.

**Methods:**

We performed a prospective, multicenter, observational study in six Spanish Intensive Care Units with 224 severe septic patients. Blood samples were collected at the time that severe sepsis was diagnosed to determine serum levels of CCCK-18, tumor necrosis factor (TNF)-alpha, interleukin (IL)-6 and IL-10. The end point was 30-day mortality.

**Results:**

Non-surviving patients (n = 80) showed higher serum CCCK-18 levels (*P<*0.001) than survivors (n = 144). Multiple logistic regression analysis showed that serum CCCK-18 levels>391 u/L were associated with 30-day survival (Odds ratio = 2.687; 95% confidence interval = 1.449–4.983; *P* = 0.002), controlling for SOFA score, serum lactic acid levels and age. Kaplan-Meier survival analysis showed that the risk of death in septic patients with serum CCCK-18 levels >391 u/L was higher than in patients with lower values (Hazard Ratio = 3.1; 95% CI = 1.96–4.84; *P<*0.001). Serum CCCK-18 levels were positively associated with serum levels of IL-6 and lactic acid, and with SOFA and APACHE scores.

**Conclusions:**

The major novel finding of our study, the largest cohort of septic patients providing data on circulating CCCK-18 levels, was that serum CCCK-18 levels are associated with mortality in severe septic patients.

## Introduction

Severe sepsis is a common, expensive, and frequently fatal condition [1], [2]. The apoptotic process is one in which cells are actively eliminated via a programmed pathway during morphogenesis, tissue remodeling, and the resolution of the immune response. Apoptosis is increased in sepsis and could contribute to multiple organ failure and death of septic patients [3]–[6].

Cytokeratin 18 (CK-18) is a protein of the intermediate filament group present in most epithelial and parenchymal cells [7]. During apoptosis CK-18 is cleaved at various sites by the action of caspases, and the resulting fragments are released into the blood [8]. Full-length CK-18 is released into the blood plasma during necrosis, and CK-18 fragments are released during apoptosis. Determination of CK-18 fragments can be carried out by using a monoclonal antibody (M30) that recognizes caspase-cleaved CK-18 fragments, containing the CK-18 Asp 396 neoepitope, without detecting native or intact CK-18 [9], [10].

Circulating levels of caspase-cleaved CK (CCCK)-18 has been studied in patients with liver [11]–[14], tumoral [15], [16] and graft-versus-host [17] diseases. However, it has scarcely been explored in septic patients [18]–[20]. These studies found higher blood CCCK- 18 levels in septic patients than in healthy controls [18]–[20]. In one study with 101 severe septic patients, higher serum CCCK-18 levels were found in non-survivors than in survivors [20]; however, the sample size was too small to demonstrate an association between serum CCCK-18 levels and early mortality and whether they could be used as a biomarker to predict outcomes in septic patients. Thus, the objective of this study was to determine whether there is an association between serum CCCK-18 levels and mortality and whether they could be used as a biomarker to predict outcomes in a large series of patients.

## Methods

### Design and Subjects

A prospective, multicenter, observational study was carried out in six Spanish Intensive Care Units between 2008–2009. The study was approved by the Institutional Ethic Review Boards of the six participating hospitals: Hospital Universitario de Canarias (La Laguna. Tenerife. Spain), Hospital Universitario Nuestra Señora de Candelaria (Santa Cruz de Tenerife. Spain), Hospital Universitario Dr. Negrín (Las Palmas de Gran Canaria. Spain), Hospital Clínico Universitario de Valencia (Valencia. Spain), Hospital San Jorge (Huesca. Spain) and Hospital Insular (Las Palmas de Gran Canaria. Spain). Written informed consent from the patients or from their family members was obtained.

A total of 224 patients with severe sepsis were included. The inclusion criteria used for severe sepsis were those defined by the International Sepsis Definitions Conference [21]. The exclusion criteria were: age <18 years, pregnancy, lactation, human immunodeficiency virus (HIV), white blood cell count <1,000/µl, solid or hematological tumor, or immunosuppressive, steroid or radiation therapy.

### Variables recorded

The following variables were recorded for each patient: sex, age, diabetes mellitus, chronic renal failure defined as glomerular filtration rate (GFR) <60 ml/min per 1.73 m^2^, chronic obstructive pulmonary disease (COPD), site of infection, microorganism responsible, bloodstream infection, empiric antimicrobial treatment, pressure of arterial oxygen/fraction inspired of oxygen (PaO_2_/FIO_2_), creatinine, bilirubin, leukocytes, lactic acid, platelets, international normalized ratio (INR), activated partial thromboplastin time (aPTT), Acute Physiology and Chronic Health Evaluation II (APACHE II) score [22] and Sepsis-related Organ Failure Assessment [SOFA] score [23].

### End-point

The end-point of the study was 30-day mortality.

### Blood samples

Blood samples from 224 patients were collected at the time severe sepsis was diagnosed. Serum was allowed to clot for 10 minutes at room temperature, then centrifuged at 1000 × g for 15 minutes and the supernatant was immediately stored in aliquot at −80°C to the end of the recruitment process. All determinations were performed by laboratory technicians blinded to all clinical data. Assays were performed at the Laboratory Department of the Hospital Universitario de Canarias (La Laguna, Santa Cruz de Tenerife, Spain).

### Serum CCCK-18 analysis

CCCK-18 levels were measured in serum by **e**nzyme-linked immunosorbent assay (ELISA) using M30 Apoptosense ELISA, PEVIVA AB (Bromma, Sweden), lot PE-0133. The M30 antibody recognises a neo-epitope exposed after caspase cleavage of K18 after the aspartic acid residue 396. M30 Apoptosense ELISA measures detects soluble caspase-cleaved K18 (ccK18) fragments containing the K18Asp396 neo-epitope. M30-antigen levels are expressed as U/l. The intra- and inter-assay coefficients of variation (CV) were <10%. The detection limit for the assay was 25 U/L.

### Serum levels of tumor necrosis factor (TNF)-alpha, interleukin (IL)-6 and IL-10 analysis

TNF-alpha, IL-6 and IL-10 were measured in serum by solid-phase chemiluminescent immunometric assays (Immulite, Siemens Healthcare Diagnostics Products, Llanberis, United Kingdom). The intra-assay CV were 3.6%, 6.2%, and 9.9%, respectively. The interassay CV were 6.5%, 7.5%, and 9.9%, respectively. The detection limits for the assays were 1.7 pg/ml, 2.0 pg/ml and 1 pg/ml respectively.

### Statistical Methods

Continuous variables are reported as medians and interquartile ranges. Categorical variables are reported as frequencies and percentages. Comparisons of continuous variables between groups were carried out using Mann-Whitney U test. Comparisons between groups for categorical variables were carried out with chi-square test. We plotted a receiver operating characteristic (ROC) curve using survival at 30 days as the classification variable, and serum CCCK-18 levels as the prognostic variable. Analysis of survival at 30 days with Kaplan-Meier method curve and comparisons by log-rank test were carried out using serum CCCK-18 levels lower/higher than 391 u/L as the independent variable, and survival at 30 days as the dependent variable. We used dot-plot to represent serum caspase-cleaved citokeratin (CCCK)-18 levels in 30-day surviving and non-surviving septic patients. Multiple logistic regression analysis was carried out to test the independent contribution of serum CCCK-18 levels higher than 391 u/L on the prediction of 30-day mortality, controlling for SOFA score, lactic acid levels and age. Odds ratio and 95% confidence intervals (CI) were calculated as measures of the clinical impact of the predictor variables. Wald test was calculated for each variable included in the regression model. We used Spearman’s rank correlation coefficient to determine the association between continuous variables. A P value of less than 0.05 was considered statistically significant. Statistical analyses were performed with SPSS 17.0 (SPSS Inc., Chicago, IL, USA) and NCSS 2000 (Kaysville, Utah).

## Results

Table 1 shows the comparison of demographic and clinical parameters between surviving (n = 144) and non-surviving (n = 80) septic patients. We found that non-surviving septic patients showed higher age, creatinine, lactic acid, INR, aPTT, SOFA and APACHE-II scores, and lower platelet count than surviving patients. In addition, non-surviving septic patients showed higher serum levels of CCCK-18 (*P<*0.001), IL-6 (*P = *0.001) and IL-10 (*P* = 0.002) than survivors. No differences were observed regarding sex, diabetes mellitus, chronic renal failure, COPD, ischemic heart disease, site of infection, microorganism responsible, bloodstream infection, antimicrobial treatment and serum levels of TNF-α.

**Table 1:** Patients’demographic and clinical characteristics.

	Survival	Non-survival	p-value
	(n = 144)	(n = 80)	
Sex male – n (%)	93 (64.6)	54 (67.5)	0.77
Age - median years (p 25–75)	55 (44–66)	64 (56–74)	<0.001
Diabetes mellitus – n (%)	40 (27.8)	31 (38.8)	0.10
Chronic renal failure – n (%)	8 (5.6)	8 (10.0)	0.28
COPD – n (%)	15 (10.4)	12 (15.0)	0.39
Ischemic heart disease - n (%)	13 (9.0)	5 (6.3)	0.61
Site of infection			0.68
Respiratory - n (%)	81 (56.3)	48 (60.0)	
Abdominal - n (%)	38 (26.4)	21 (26.3)	
Neurological	3 (2.1)	0	
Urinary - n (%)	8 (5.6)	4 (5.0)	
Skin - n (%)	8 (5.6)	3 (3.8)	
Endocarditis - n (%)	6 (4.2)	4 (5.0)	
Microorganism responsibles			
Unknwon - n (%	75 (52.1)	43 (53.8)	0.89
Gram-positive- n (%)	33 (22.9)	21 (26.3)	0.63
Gram-negative- n (%)	35 (24.3)	16 (20.0)	0.51
Fungii- n (%)	4 (2.8)	4 (5.0)	0.46
Anaerobe- n (%)	1 (0.7)	1 (1.3)	0.99
Bloodstream infection - n (%)	22 (15.3)	11 (13.8)	0.85
Empiric antimicrobial treatment adequate			0.96
Unknown due to negative cultures- n (%)	75 (52.1)	43 (53.8)	
Adequate - n (%)	58 (40.3)	30 (37.5)	
Unknown due to antigenuria diagnosis-n(%)	4 (2.8)	3 (3.8)	
Inadequate- n (%)	7 (4.9)	4 (5.0)	
Betalactamic plus aminoglycoside - n (%) (%)aminoglycoside- n (%)	29 (20.1)	20 (25.0)	0.40
Betalactamic plus quinolone - n (%)	79 (54.9)	43 (53.8)	0.89
Pa0_2_/FI0_2_ ratio - median (p 25–75)	170 (113–262)	180 (100–244)	0.38
Creatinine (mg/dl) - median (p 25–75)	1.20 (0.80–1.95)	1.50 (0.90–2.75)	0.02
Bilirubin (mg/dl) - median (p 25–75)	0.90 (0.44–1.53)	1.00 (0.47–2.44)	0.60
Leukocytes (cells/mm^3^) - median*10^3^ (p 25–75)	15.2 (10.0–20.7)	15.3 (9.4–21.3)	0.88
Lactic acid (mmol/L) - median (p 25–75)	1.80 (1.05–3.50)	3.50 (1.45–5.95)	<0.001
Platelets (cells/mm^3^) - median*10^3^ (p 25–75)	192 (130–273)	124 (76–222)	<0.001
INR - median (p 25–75)	1.27 (1.10–1.53)	1.42 (1.16–1.90)	0.01
aPTT (seconds) - median (p 25–75)	32 (28–42)	38 (29–46)	0.01
SOFA score - median (p 25–75)	9 (7–11)	11 (9–15)	<0.001
APACHE-II score - median (p 25–75)	18 (14–22)	23 (18–28)	<0.001
CCCK-18 (U/l) - median (p 25–75)	311 (230–443)	453 (311–711)	<0.001
TNF-alpha - median pg/ml (percentile 25–75)	31.8 (20.0–51.2)	39.4 (18.7–76.8)	0.29
Interleukin-6 - median pg/ml (percentile 25–75)	104 (42–578)	504 (58–1000)	0.001
Interleukin-10 median pg/ml (percentile 25–75)	10.4 (5.7–38.0)	52.0 (8.4–162.5)	0.002

COPD = Chronic Obstructive Pulmonary Disease; PaO_2_/FIO_2_ = pressure of arterial oxygen/fraction inspired oxygen; aPTT = Activated partial thromboplastin time; INR = International normalized ratio; Acute Physiology and Chronic Health Evaluation (APACHE)-II score; SOFA = Sepsis-related Organ Failure Assessment; CCCK = caspase-cleaved citokeratin; TNF = tumor necrosis factor (TNF)-alpha; data are presented as number (percentage) or median (interquartile range).

Multiple logistic regression analysis showed that serum CCCK-18 levels>391 u/L were associated with 30-day survival (Odds ratio = 2.687; 95% CI = 1.449–4.983; *P* = 0.002), controlling for SOFA score, serum lactic acid levels and age (Table 2).

**Table 2:** Multiple logistic regression analyses to predict survival at 30 days.

	Wald test	Odds Ratio	95% Confidence Interval	*P*-value
Serum CCCK-18 levels>391 u/L	9.83	2.687	1.449–4.983	0.002
SOFA score	6.45	1.125	1.027–1.232	0.01
Serum lactic acid levels (mmol/L)	2.98	1.108	0.986–1.246	0.08
Age (years)	5.61	1.027	1.005–1.050	0.02

CCCK = caspase-cleaved citokeratin; SOFA = Sepsis-related Organ Failure Assessment.

**Figure 1:**
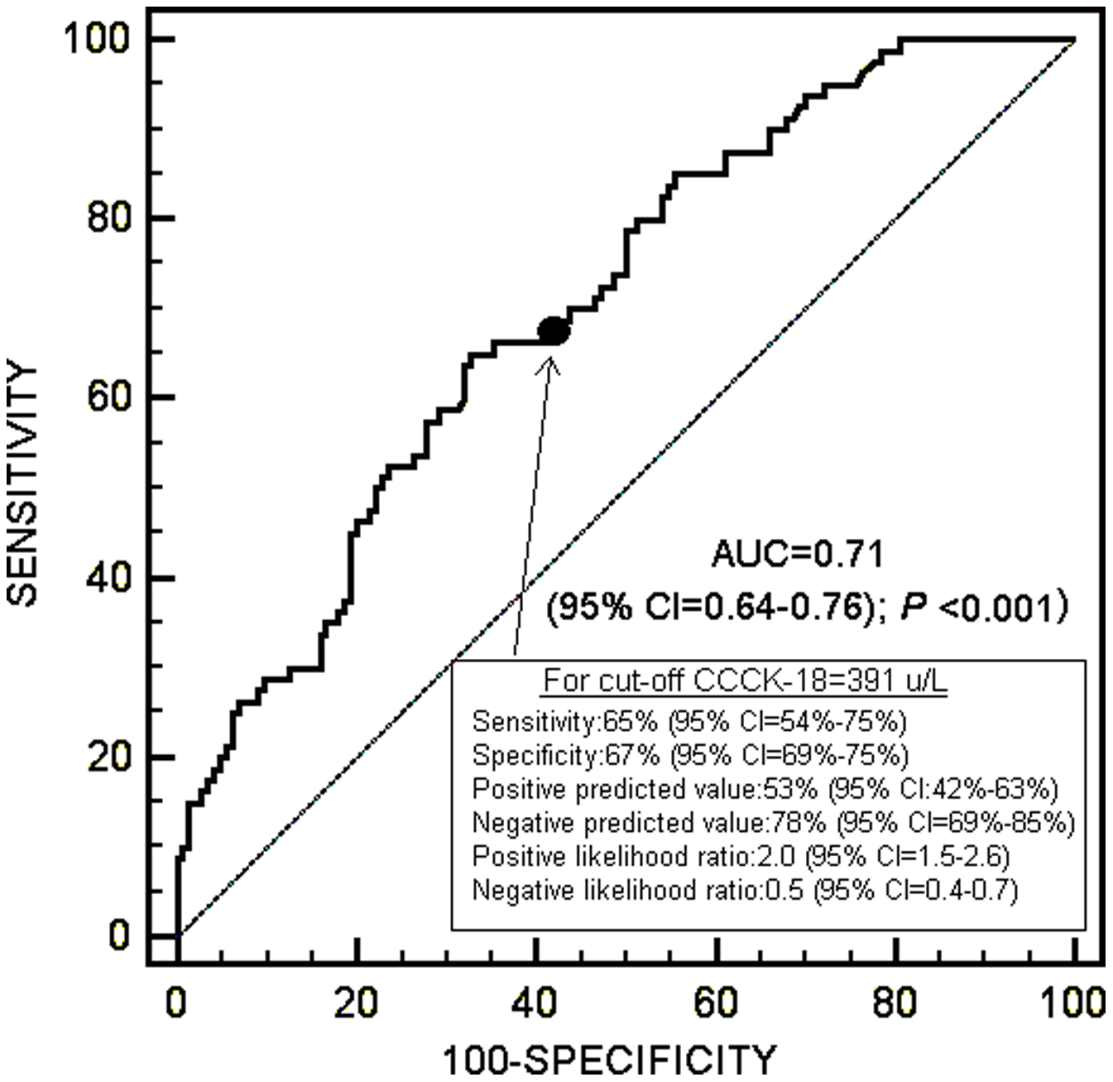
Receiver operation characteristic analysis using serum caspase-cleaved cytokeratin (CCCK)-18 levels as predictor of mortality at 30 days.

Receiver Operating Characteristic (ROC) analysis showed that the area under curve of serum CCCK-18 levels to predict 30-day survival was 0.71 (95% CI = 0.64–0.76; *P<*0.001) (Figure 1).

**Figure 2:**
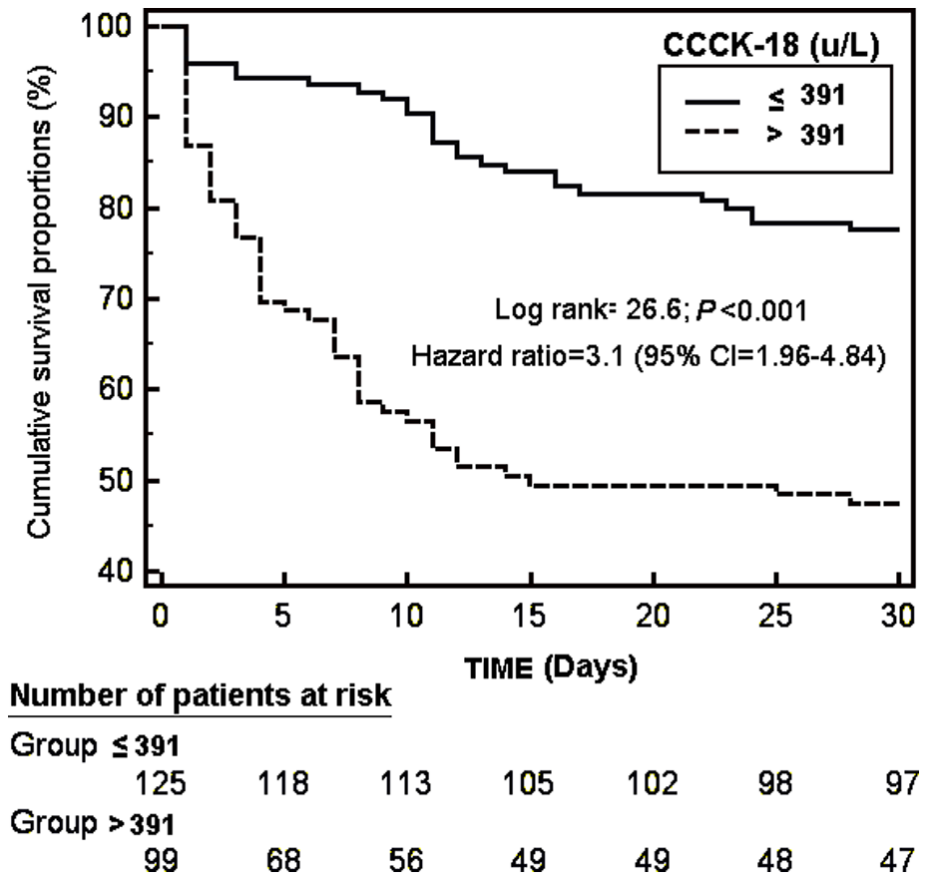
Survival curves at 30 days using serum caspase-cleaved citokeratin (CCCK)-18 levels higher or lower than 391 u/L.

Kaplan-Meier survival analysis showed that the risk of death in septic patients with serum CCCK-18 levels above 391 u/L was higher than in patients with lower values (Hazard Ratio = 3.1; 95% CI = 1.96–4.84; *P<*0.001) (Figure 2).

**Figure 3:**
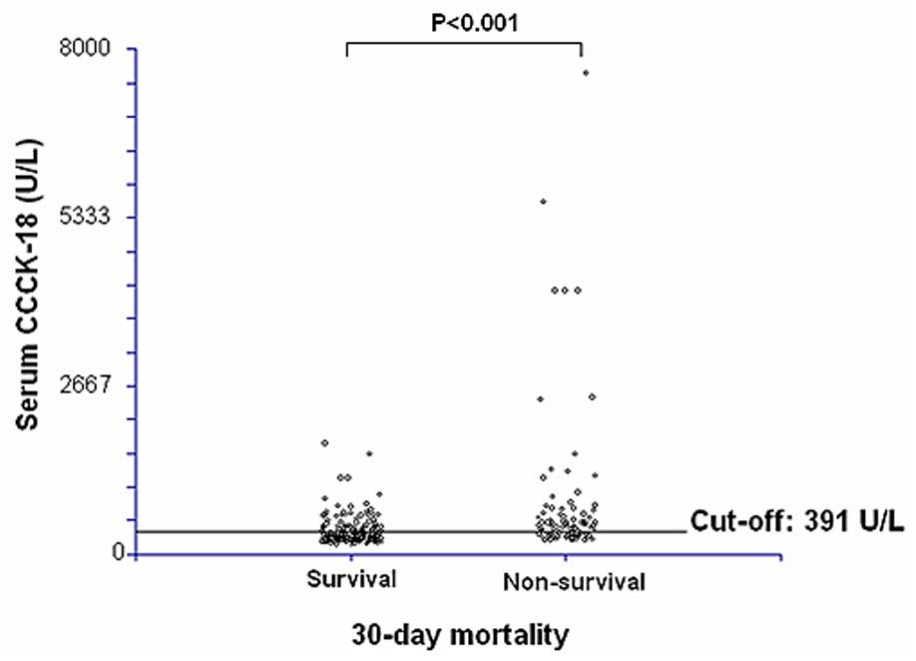
Dot-plot of serum caspase-cleaved citokeratin (CCCK)-18 levels in 30-day surviving and non-surviving septic patients.

We ploted serum caspase-cleaved citokeratin (CCCK)-18 levels in 30-day surviving and non-surviving septic patients (Figure 3).

We found that survival patients at 30 days with serum CCCK-18 levels above 391 u/L at the time that severe sepsis was diagnosed showed higher ICU stay that patients with lower values [24 (12–46) vs 15 (7–31) days; p = 0.04].

Serum CCCK-18 levels were positively associated with serum levels of IL-6 and lactic acid, and with SOFA and APACHE scores (Table 3).

**Table 3:** Correlation of serum caspase-cleaved citokeratin (CCCK)-18 levels with serum levels of TNF-α, interleukin-6, interleukin-10, and lactic acid, and with SOFA and APACHE-II scores.

	rho	*P*-value
TNF-α	0.09	0.31
Interleukin-6	0.15	0.03
Interleukin-10	0.11	0.21
Lactic acid (mmol/L)	0.20	0.003
SOFA score	0.27	<0.001
APACHE score	0.30	<0.001

TNF = tumor necrosis factor; SOFA = Sepsis-related Organ Failure Assessment score**;** APACHE = Acute Physiology and Chronic Health Evaluation (APACHE)-II score; rho = Spearman’s rank correlation coefficient.

We found that patients with liver dysfunction showed higher serum CCCK-18 levels than patients without it [413 (278–626) vs 327 (251–500) U/l; p = 0.01], and that patients with kidney dysfunction showed higher serum CCCK-18 levels than patients without it [398 (280–628) vs 318 (237–450) U/l; p<0.001].

## Discussion

To our knowledge, this study includes the largest series providing data on circulating CCCK-18 levels in septic patients. The major novel findings of our study were that serum CCCK-18 levels are associated with mortality and could be used as a biomarker to predict outcomes in septic patients.

We found higher serum CCCK-18 levels in non-surviving septic patients than in survivors. These findings are in consonance with those of a study by Hofer et al. with 101 severe septic [20]; however, the sample size in that study was too small to demonstrate whether serum CCCK-18 levels are associated with mortality. The greater sample size of our study enabled us to carry out multiple regression analysis and showed, for the first time, that serum CK-18 levels are associated with mortality in septic patients. The advantage of measuring serum CCCK-18 levels to predict survival at 30 days compared with SOFA score underlies in a higher predictive value measured with the Wald test.

Another interesting new finding of our study, according to the results of ROC curve analysis, was that serum CK-18 levels could be used as a biomarker to predict outcomes in septic patients.

Hofer et al. found an association between serum CCCK-18 and lactate levels [20]. In our study, we also found this association. In addition, we found an association between serum CCCK-18 levels and other measures of sepsis severity, such as SOFA and APACHE-II scores for the first time.

Taken together, these results indicate that serum CCCK-18 levels may be of great pathophysiological significance in septic patients and that apoptosis could contribute to multiple organ failure and death of septic patients. Apoptotic cell death occurs primarily through three different pathways: the extrinsic death receptor pathway (type I cells), the intrinsic (mitochondrial) pathway (type II cells) and the endoplasmic reticulum or stress-induced pathway [3]–[6]. In type I cells, apoptosis is initiated by the activation of a surface death receptor of tumor necrosis factor receptor superfamily (TNFRSF) by its cognate death ligand (TNFSF). In type II cells, the initiation of apoptosis can be activated by cytokines such as interleukin (IL)-1 and IL-6, oxygen free radicals and nitric oxide (NO); and could be reduced by the activation of anti-apoptotic members of the Bcl-2 family by means of IL-10. The death signal is transduced, then a cascade of caspases leads to cell death. During this caspase activation, CK-18 is cleaved and CCCK-18 is released into the blood. In our study, we found an association between serum CCCK-18 and Il-6 levels; however, we did not find an association between serum CCCK-18 and TNF-alpha and IL-10 levels.

From a therapeutic perspective, the development of modulators of apoptotic activity could be used as a new class of drugs for the treatment of severe sepsis. In septic rats, the administration of modulators of apoptotic activity has been found to reduce apoptosis and increase survival rates [24]–[26].

The strengths of our study are that it was a multicenter study (which increases the external applicability of results to other similar units) and the large sample size (which allowed us to carry out multiple logistic regression analysis to determine the association between serum CCCK-18 levels and mortality). However, some limitations of our study should be recognized. First, no analysis of serum CCCK-18 levels during follow-up was performed. Second, measuring other inducers of apoptosis and compounds of apoptosis would be desirable in order to better evaluate the relationship with these aspects. Third, we did not determine serum CCCK-18 levels in control groups, such as patients with non-infectious systemic inflammatory response syndrome, and this would have been interesting to assess whether CCCK-18 measurements might be useful for early diagnosis of sepsis. Four, the determination of total serum CCCK-18 levels by M65 ELISA or M65 EpiDeath ELISA would have been interesting in order to quantify the leading mode of cell death (apoptosis or necrosis).

## Conclusions

The major novel finding of our study, the largest cohort of septic patients providing data on circulating CCCK-18 levels, was that serum CCCK-18 levels are associated with mortality in severe septic patients.
